# Intussusception revealing right colonic adenocarcinoma in a 61-year-old woman: a case report

**DOI:** 10.1186/s13256-023-04027-4

**Published:** 2023-07-14

**Authors:** Mohamed Osama Mohamed Ali, Noon Idris Abdelrahman Mohamed, Ahmed Abdelfattah Eltomelhussein Ahmed, Mohamed Osman Suliman Basher, Samya Abbas Abdelrazig Mohamed, Osama Mohieldin Elgemaabi

**Affiliations:** Department of General Surgery, Military Hospital, Khartoum, Sudan

**Keywords:** Colicky pain, Intussusception, Colon, Right iliac fossa mass, Case report

## Abstract

**Introduction:**

Adult Intussusception is an uncommon diagnosis, with one to three cases occurring in a population of 1,000,000 per year, primarily due to underlying pathological lead points, of which 70% are malignant. Lipoma is the most common benign tumour, and primary adenocarcinoma is the most common malignant one. Early diagnosis and treatment are essential to reducing poor outcomes, including ischemia, perforation, and sepsis. Computed tomography imaging is a modality of choice for diagnosis. With a diagnostic accuracy of up to 100% and a specificity of up to 71%. Surgical intervention is the definitive treatment, and the decision is taken according to the situation.

**Case presentation:**

A 61-year-old Black African female presented to our surgical casualty with intermittent colicky abdominal pain for 1 month. After that, she started to experience abdominal swelling in the right iliac fossa. A CT scan confirmed the presence of colo-colic Intussusception, cecum-ascending-transverse colon. Laparotomy was scheduled, and a right hemicolectomy was done accordingly. Diagnosis of adenocarcinoma (Dukes stage B2) was made histologically.

**Conclusion:**

Intussusception in adults is a challenging diagnosis requiring high clinical suspicion and has a high incidence of fatal complications. Colonic cancer can be worsening by any infection and chronic medical problem. CT imaging is the lifesaving modality of choice for diagnosis. Good patient outcomes depend on timely diagnosis and recruitment of a multi-disciplinary team.

## Introduction

Intussusception means telescoping of a proximal segment of the bowel into the lumen of the adjacent distal segment (intussuscipiens). Rarely the opposite could happen, which is known as retrograde Intussusception [1].

Intussusception is very uncommon in adult patients, with one to three cases occurring in a population of 1,000,000 per year, which puts it at the bottom of the list of differential diagnoses of abdominal pain. More often, it may not be counted. Its aetiology differs significantly between children and adults. Adult Intussusception of the colon is most likely secondary to a malignant tumour [2].

In both small- and large-bowel Intussusception, the lipoma is the most common benign tumour, and adenocarcinoma is the common malignant leading point [3].

The diagnosis is commonly surprised at laparotomy, as most patients present as an emergency with intestinal obstruction. Usually, in stable patients, the diagnosis can be challenging as symptoms, mainly intermittent abdominal pain and clinical examination and investigations are often negative, and the patient will probably be labelled with irritable bowel syndrome [4].

Colonic cancer can be worsen by any infection and chronic medical problem [5].

This work has been reported in line with the CARE criteria [6].

## Case presentation

A 61-year-old Black African female, medically free, presented to the emergency department of a Military hospital in Khartoum, Sudan, with severe colicky abdominal pain and right iliac fossa swelling for 1 month. The pain was sudden onset, severe, intermittent, and colicky in the right iliac fossa, preventing her from doing her daily activities. It was localized, not radiating to other areas, aggravated by food intake and not relieved by analgesics. On further questioning, it was revealed that there were streaks of blood in her stool. However, there was no constipation, diarrhoea, or vomiting. The patient was experiencing loss of appetite and unintentional weight loss, about 15 kg, 3 months before her illness. Body mass index was 24.2. There was no fever, night sweats, or cardiopulmonary complaints. The patient has no history of a similar condition and had not been diagnosed with colon cancer, inflammatory bowel disease, irritable bowel disease or any recent evidence of pulmonary tuberculosis infection.

She has no family history of colon cancer; however, she has a history of breast cancer in the family. At the time, she was not on any medication or had any known allergies. She is a non-smoker and non-alcoholic.

On physical examination, the patient looks ill, conscious, vitally stable, not pale nor jaundiced and has no signs of dehydration.

Abdominal examination revealed a right iliac fossa mass, oval in shape, 12 × 10 cm, irregular surface, normal skin over it and no scars. The mass has a normal temperature, is tender, and is hard in consistency. It has well-defined edges, is mobile, not compressible or reducible, and not pulsatile. All hernia orifices are intact, and no lymph node enlargement. However, the rest of the abdominal examination was unremarkable, and the digital rectal examination was normal. Abdominal ultrasound was ordered and revealed the presence of colonic mass and multiple gall stones, but it was otherwise not remarkable.

**Fig. 1:**
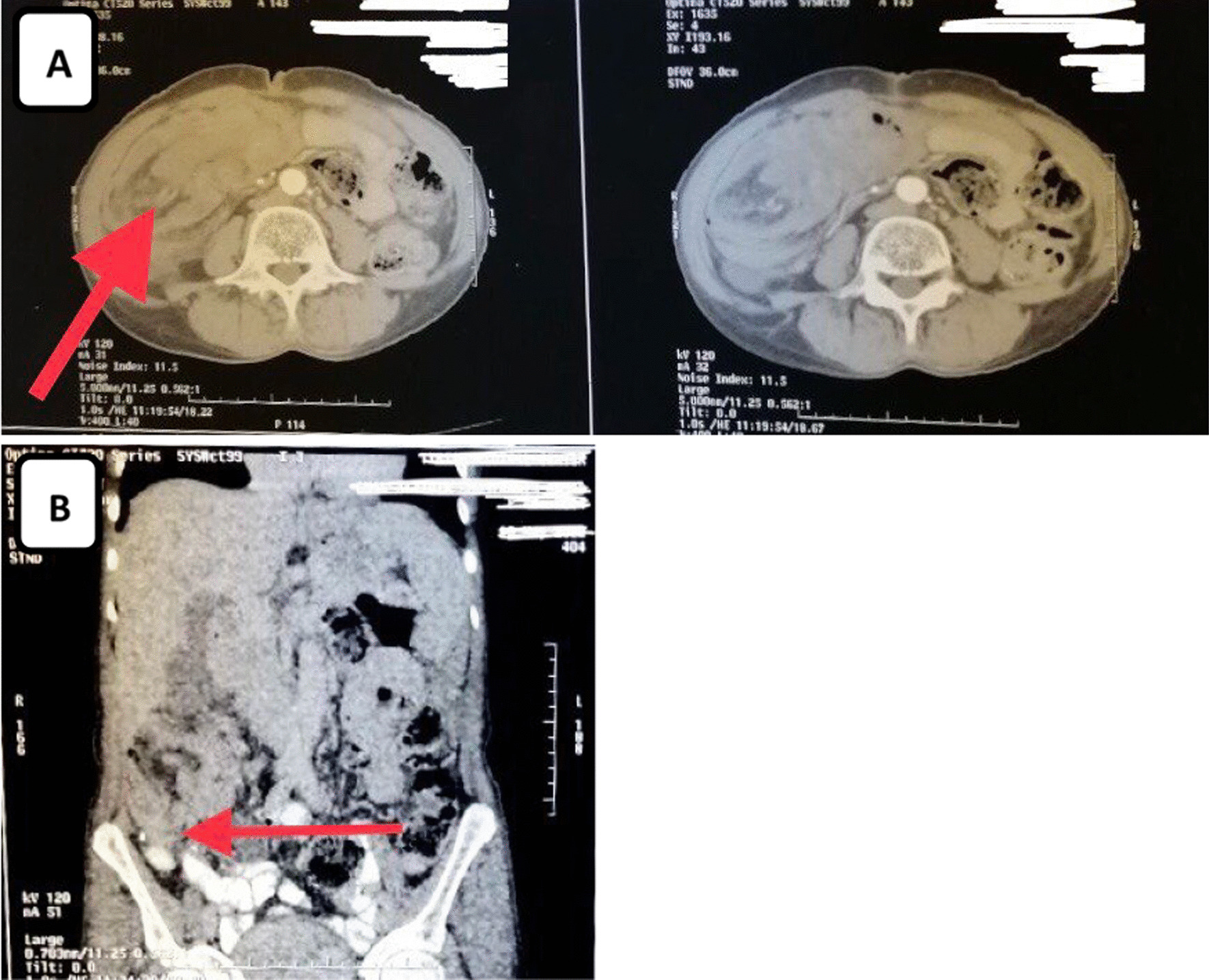
**A** Axial computed tomography of the abdomen shows the thickened wall of the intussuscipiens and the mass around the invaginating mesenteric fat (target sign) (the red arrow). **B** Coronal section of abdominal computed tomography shows the cecal mass (the red arrow) and thickened intestinal wall

Then computed tomography imaging abdomen was ordered and that showed a segment of bowel loop was seen invaginated into its proximal segment with bowel wall thickening in the ascending colon extending to the transverse colon, which confirmed the diagnosis of colo-colic intussusception. Also, multiple gall stones were elicited by the CT scan (Fig. 1).

The patient has low serum sodium, potassium, total bilirubin and albumin levels. Serum CEA tumour marker was within normal ranges. Chest x-ray and echocardiography were normal.

**Fig. 2:**
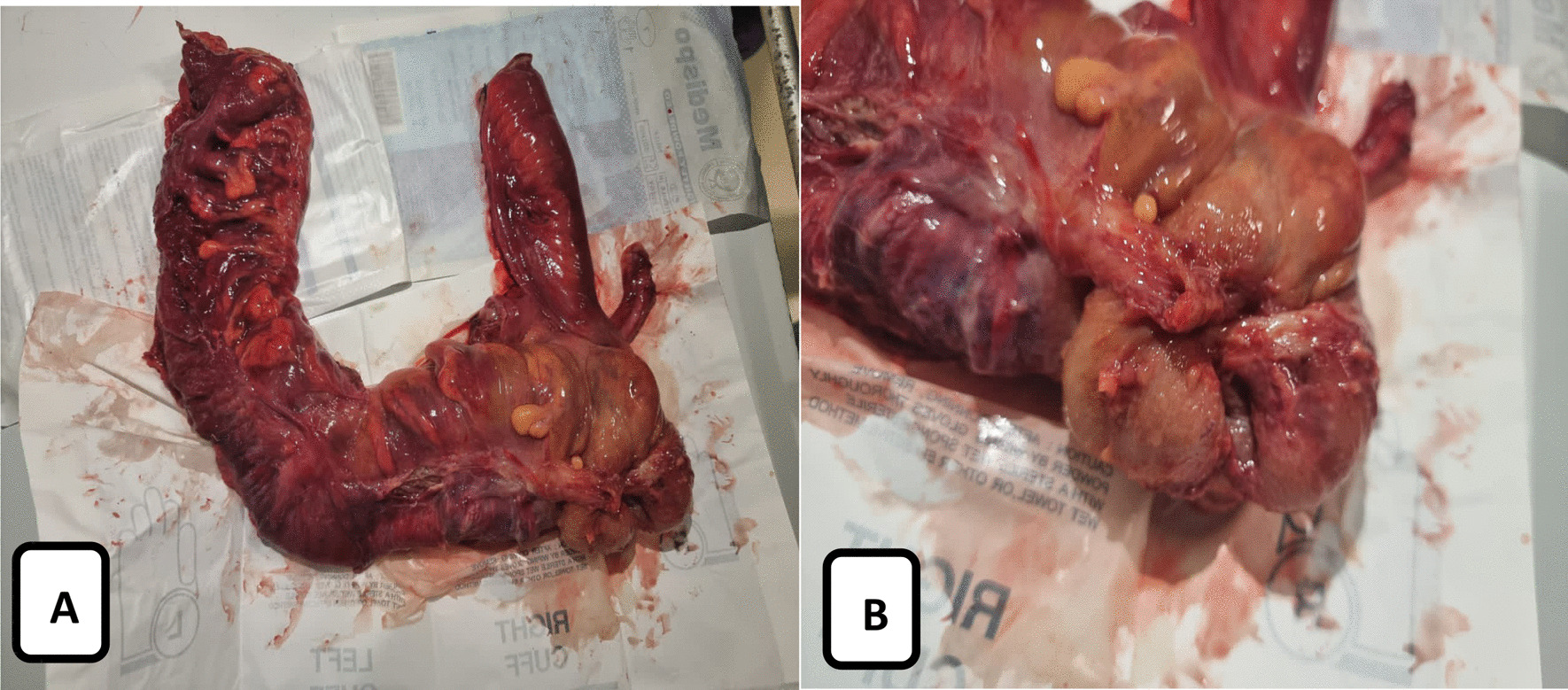
**A** Shows the resected bowel specimen. **B** Shows the externally visible cecal mass

So, an elective laparotomy was scheduled 2 days after the presentation. The patient was kept nil per mouth with full fluid maintenance, antibiotic, proton pump inhibitor, and prophylactic anticoagulant. An incision abdomen was opened through a midline, and a large mass was found, including the terminal ileum, the ileocecal valve, the appendix, the cecum and the ascending colon, which were invaginated in the transverse colon. Adhesions were found between the abdominal wall and the mass and were released, as colonic milking was unsuccessful. The surgeon decided to go for the right hemicolectomy and end-to-end anastomosis (Fig. 2).

**Fig. 3:**
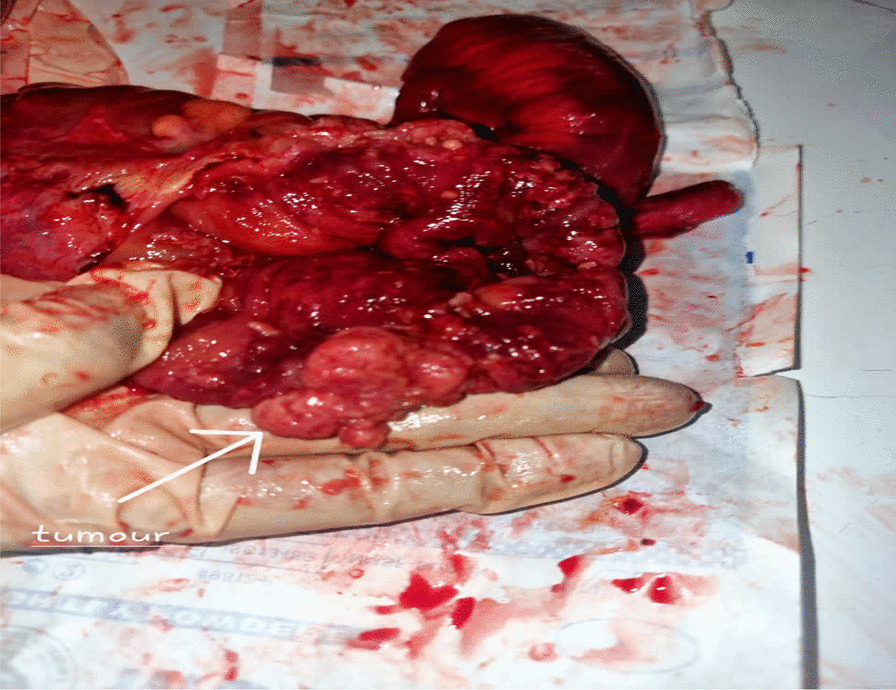
Specimen of resected cecum cut section showing lobulated mass, which is the leading point of intussusception

The cut section of the cecum showed a fungating mass of 6 × 5 × 3 cm (Fig. 3).

The histopathological assessment confirmed the diagnosis of well-differentiated grade one adenocarcinoma with perforated muscularis mucosa only, Dukes stage B2. No evidence of lymph-vascular invasion. Furthermore, all the examined lymph nodes were free of neoplasm.

No complications were encountered post-operatively. The patient was discharged home after 5 days and seen after 2 weeks in the clinic in good condition. No evidence of synchronous colorectal cancer was observed by colonoscopy. Oncological follow-up was launched immediately and planned for chemotherapy. One month later the patient was seen in the clinic in a good condition clinically and all investigations lie within normal ranges.

## Discussion

Intussusception happens when the proximal segment of the bowl telescope into the adjacent loop, causing obstruction and affecting the blood supply, which could lead to intestinal obstruction, ischemia, perforation and sepsis. Intussusception has a variety of types according to the involved segments [7].

It is more common to be a pediatric presentation than in adults, found in less than 1 in 1300 abdominal operations [8].

In adults, its aetiology is always related to neoplasm as a lead point in nearly 90% of cases. Malignant tumours cause approximately 60–80% of intussusceptions in the large bowel [4]. So if it is predicted by imagining, the suspicion of tumours is the top priority, as in our case.

Other risk factors that lead to an intussusception may include:

- Mass (benign or malignant)
- Anatomical changes
- Post-surgical adhesions
- Endometriosis
- Idiopathic
- Fibroids
- Gastrostomy tube
- Jejunostomy tube [9].

In a previous study, the lesions were found to be varied from 3.5 cm to 8.5 cm in diameter [10].

Inflammatory diseases of the colon or appendix can also play a leading point in Intussusception.

Symptoms are sometimes vague, including colicky abdominal pain, intermittent or constant vomiting (can be bilious), bloating, and bloody stool [11].

Inpatient presented with non-emergency nonspecific abdominal pain. CT scan appears to be the most sensitive diagnostic tool for picking Intussusception, with a diagnostic accuracy of 58–100% and a specificity of 57–71% [12].

The CT findings one can illustrate will be a mass-like lesion, including the inner intussusceptum, an eccentric fat density mass that represents the intussuscepted mesenteric fat, and the outer intussuscipiens, and this appears as a “target” or a “sausage” mass according to the imaging plane.

Additionally, a CT scan can aid in identifying the pathological lead points, and its extension also could help in anticipating the vascular status of the bowel. In some circumstances, a CT scan can predict the possibility of self-resolution of the condition [7].

In our case, the abdominal CT scan picks up the colo-colic Intussusception and the colonic mass extension that sharply changes the forward workup of the patient.

Ultrasound is also a helpful tool but less sensitive than CT scans. The characteristic features that could be revealed by ultrasound include target and doughnut signs in transverse view and pseudo-kidney signs in longitudinal view [13].

The ultra-sonographic findings at the first presentation of our patient raised the colonic mass suspension, so an immediate CT scan was ordered. However, it did not elicit the presence of Intussusception.

Surgical interventions are the mainstay of management of adult intussusception as it carries a very high incidence of underlying malignancy. After supportive emergency management, preoperative preparation of the patient for resection according to the appropriate oncological assessment is the plan [13].

Intraoperatively, the location, size, and cause of the Intussusception and the viability of the bowel determine the appropriate decision for the surgical procedure [14].

As the management is purely surgical in adults, there is still controversy about the trail of reduction intraoperatively before resection. There is a debate between two opposing schools. One supports intraoperative reduction as it may minimise unnecessary bowel resection. Another school is fighting intussusception reduction as the risk of dissemination of the malignant cells during the procedure [15].

Adult Intussusception carries a poor prognostic picture due to the delay in diagnosis of its nonspecific presentation and the prevalence of underlying malignancy. This return the complications of vascular supply to be jeopardised and sepsis to manifest earlier with a high mortality rate, especially in developing settings such as Sudan [12].

## Conclusion

Intussusception in adults is a challenging diagnosis requiring high clinical suspicion and has a high incidence of fatal complications. CT imaging is the lifesaving modality of choice for diagnosis.

Good patient outcomes depend on timely diagnosis and recruitment of an interprofessional team.

## Data Availability

The datasets used and/or analysed during the current study are available from the corresponding author on reasonable request.
